# Comprehensive approach to costing cervical cancer prevention and control: a case study in the United Republic of Tanzania using the Cervical Cancer Prevention and Control Costing (C4P) tool

**DOI:** 10.1186/s12916-022-02576-x

**Published:** 2022-11-01

**Authors:** Ann Levin, Safina Yuma, Edwin Swai, Winthrop Morgan, Cindy L. Gauvreau, Nathalie Broutet, Karene Hoi Ting Yeung, Raymond Hutubessy

**Affiliations:** 1 Levin & Morgan LLC, Bethesda, USA; 2 Ministry of Health, Dodoma, United Republic of Tanzania; 3 World Health Organization, Dar es Salaam, United Republic of Tanzania; 4 Child Health Evaluative Sciences, The Hospital for Sick Children, Toronto, Canada; 5 Department of Reproductive Health and Research, World Health Organization, Geneve, Switzerland; 6 Department of Immunization, Vaccines and Biologicals, World Health Organization, Geneva, Switzerland

**Keywords:** Costing, Cervical cancer, Human papillomavirus (HPV), Vaccines, Immunization programmes, Screening, Treatment, United Republic of Tanzania

## Abstract

**Background:**

The World Health Organization (WHO) has developed a costing tool, the Cervical Cancer Prevention and Control Costing (C4P) tool, to estimate the comprehensive cost of cervical cancer primary, secondary and tertiary prevention in low- and middle-income countries. The tool was piloted in the United Republic of Tanzania, a country with a high incidence of cervical cancer with 62.5 cases per 100,000 women in 2020. This paper presents the costing tool methods as well as the results from the pilot in Tanzania.

**Methods:**

The C4P tool estimates the incremental costs of cervical cancer prevention and control programmes. It estimates the financial (monetary costs to the government) and economic costs (opportunity costs). For the pilot, the study team collected data on costs and programme assumptions for human papillomavirus (HPV) vaccination of 14-year-old girls and scaling up of cervical cancer screening (visual inspection with acetic acid and HPV-DNA testing) and treatment for women for 2020–2024. Assumptions were made on how vaccination coverage would increase over the 5 years as well as developing additional screening and treatment capacity through health personnel training and infrastructure strengthening.

**Results:**

The total financial and economic costs of the comprehensive programme during 2020–2024 are projected to be US$68 million and US$124 million, respectively. The financial and economic costs of a fully immunized girl with HPV vaccine are estimated to be US$6.68 and US$17.31, respectively, while the costs per woman screened for cervical cancer are, on average, US$4.02 and US$5.83, respectively; US$6.44 and US$9.37 for pre-cancer treatment, respectively; and US$101 and US$107 for diagnosis of invasive cancer, respectively. The cost of treating and managing invasive cancer range from US$7.05 and US$7.83 for outpatient palliative care to US$800.21 and US$893.80 for radiotherapy, respectively.

**Conclusions:**

The C4P costing tool can assist national cervical cancer programmes to estimate monetary resources needed as well as opportunity costs of reducing national cervical cancer incidence through primary, secondary and tertiary prevention.

## Background

Cervical cancer is the fourth most common cancer among women globally with 604,127 annual new cases and 341,831 deaths in 2020 [1]. The World Health Organization has launched a global strategy to accelerate countries to eliminate cervical cancer as a public health problem by the end of this century [2]. This strategy proposes to reduce cervical cancer incidence and mortality through reaching by 2030 and maintaining along the years the following targets: 90% coverage for the full course of human papillomavirus (HPV) vaccination for girls by 15 years of age, 70% screening coverage of eligible women twice in their lifetime by 35 and 45 years of age with a high-performance test and provision of treatment and management care for 90% of women with pre-cancer and invasive cancer.

To assist countries to introduce programmes to prevent and control cervical cancer, the World Health Organization developed the Cervical Cancer Prevention and Control Costing (C4P) tool [3] for the estimation of financial and economic costs over 5 years. The tool has two modules—one for estimating the incremental costs of introducing HPV vaccination (primary prevention) and the second for estimating the incremental costs of cervical cancer screening and treatment (secondary and tertiary prevention). An earlier paper [4] presented the assumptions and methods used for the first module. This paper will present the assumptions and methods used in the second module and the improvement in the first module. It will also present the overall estimated incremental costs of introducing vaccination and screening and treatment in Tanzania.

Tanzania had the fourth highest incidence rate of cervical cancer in the world in 2020 with 62.5 new cases per 100,000 women (age-standardized to the world population) [5]. Cervical cancer mortality is also high, with 42.7 deaths per 100,000 (age-standardized to the world population) in 2020 [5]. Tanzania’s high incidence of cervical cancer is linked to the burden of human immunodeficiency virus/acquired immunodeficiency syndrome (HIV/AIDS) in the country, with the HIV prevalence of female adults aged 15–49 at 6.0% in 2020 [6].

The Ministry of Health of Tanzania plans to reduce cervical cancer incidence and mortality [7] and reach elimination through scaling up prevention and control activities in the country. To inform domestic and external financial resource mobilization, the objective of this study is to estimate the financial and economic incremental cost of scaling up coverage of primary, secondary and tertiary cervical cancer prevention in Tanzania from 2020 to 2024. This study is a follow-up to the findings presented in an earlier 2012 publication [4] where the costs of introducing HPV vaccination in Tanzania were projected for the years 2011 to 2015. This study differs from the earlier one since it takes a comprehensive approach to prevent and control cervical cancer and includes projected costs of scaling up cervical cancer secondary and tertiary interventions in addition to vaccination as part of the global cervical cancer elimination strategy [2].

## The Tanzanian cervical cancer prevention and control programme

The Tanzanian government has programmes to implement primary, secondary and tertiary prevention for cervical cancer as shown in Table 1. The country through the national Immunization and Vaccine Development Programme (IVD) introduced HPV vaccination on a pilot basis in 2014 and then scaled up nationally as part of the routine programme in 2018. The IVD provides two doses to girls aged 14 years through the routine programme annually with two service delivery strategies: (1) vaccination where girls visit health facilities to get the immunization and (2) vaccination by health workers who visit schools located near facilities to immunize all 14-year-old girls. It is assumed that the vaccine coverage in 2020 for 14-year-old girls would be 70% in 2020, and the coverage will increase to 85% by 2024.

**Table 1:** Implementation strategies for cervical cancer prevention and control in Tanzania, 2020–2024

	Target population	Intervention	Sites providing services
Vaccination	Girls aged 14 years	2 doses human papillomavirus vaccine [8]	Health centres, dispensaries, schools
Screening	Women living with human immunodeficiency virus: 15–49 years Women of the general population: 30–49 years	Visual inspection with acetic acid HPV-DNA	Health centres, dispensaries, all hospitals^a^ Regional referral and district hospitals
Pre-cancer treatment	Women with a positive screening test	Cryotherapy Loop electrical excision procedures	Health centres, dispensaries, regional referral and district hospitals All hospitals
Tertiary interventions for cancer	Screened women suspicious of cancer	Diagnostics Chemotherapy Radiotherapy Palliative care	All hospitals^a^ Regional referral hospitals Regional referral hospitals Health centres, regional referral and district hospitals

^a^All hospitals include zonal, regional referral and district hospitals

The National Tanzanian Reproductive Health Programme is managing the cervical cancer secondary and tertiary prevention service provision. It oversees screening with visual inspection with acetic acid (VIA) services in 136 out of 169 districts at zonal, regional referral and district hospitals and some health centres and dispensaries. By 2019, the programme had screened 11% of its eligible women. The programme managers plan to gradually ramp up VIA screening capacity from 794 in 2020 to all of its 6447 facilities by 2024. They also plan to scale up HPV-DNA screening from all 30 regional referral hospitals beginning in 2020 to 88 facilities in 2024, including 58 district hospitals.

Women with a positive VIA or HPV screening test are treated with cryotherapy, if eligible, at the same visit in a single visit approach strategy (SVA) for small lesions and loop electrical excision procedures (LEEP) for larger lesions. Health providers offer cryotherapy at all regional referral hospitals, half of the district hospitals and health centres and some dispensaries (20%). LEEP is provided at all zonal and regional referral hospitals and a quarter of district hospitals.

Women who have lesions and are suspicious of cancer are referred for further evaluation and diagnosis with colposcopy and biopsies for histopathology assessment. If they are diagnosed with invasive cervical cancer, they are referred for treatment appropriate to the stage of the cancer, including radiotherapy, chemotherapy and/or palliative care. No surgery was available in the country for the treatment of invasive cancers. Treatment is provided at a few centres in the country—two regional referral hospitals for radiotherapy and 33 for chemotherapy. Outpatient palliative care is given at regional referral and district hospitals and health centres while inpatient palliative care is given at regional referral hospitals.

## Methods

The C4P tool [3] is an Excel-based country-specific costing and planning tool that facilitates data collection and enables the user to estimate the value of incremental (additional) resources required to add the countrywide delivery of HPV vaccine to an existing immunization programme over a 5-year period and the resources required for scaling up cervical cancer screening and treatment services over a 5-year period. In other words, it only estimates the value of additional resources needed to introduce the vaccine or cervical cancer services and does not include the cost of the existing immunization or reproductive health programme or other goods and services (for example, transport) already being used for other vaccines (shared costs). The C4P tool separates costs into recurrent and capital as well as financial (monetary outlays) and economic (opportunity costs) ones. Recurrent costs are the value of resources that last less than 1 year, such as supplies and personnel time, while capital costs are the value of resources that last more than 1 year, such as equipment or vehicles (see Table 2). Startup costs are the value of resources used in the initial one-time programmatic activities such as initial training and planning and are treated as capital goods and depreciated if their useful life lasts more than a year.

**Table 2:** Activities for HPV vaccination and cervical cancer screening and treatment costed in the C4P tool

	HPV vaccination	Cervical cancer screening and treatment
**Startup costs**^a^	Microplanning Training Sensitization/social mobilization	Programme planning/microplanning Training Sensitization/social mobilization/communication
**Recurrent** Service delivery Programmatic	Vaccine procurement Personnel time Per diems and travel allowances Monitoring and supervision	Procurement of supplies Personnel time Laboratory tests Monitoring and supervision
**Capital**	Cold chain Other capital goods	VIA and HPV-DNA equipment Cryotherapy machines LEEP machine and other equipment Radiotherapy treatment

*DNA* deoxyribonucleic acid, *HPV* human papillomavirus, *LEEP* loop electrosurgical excision procedure, *VIA* visual inspection with acetic acid

^a^Designated as capital costs if one-time cost and useful life is more than 1 year

Financial costs are the monetary outlays paid for HPV vaccination or cervical cancer services such as supplies and commodities. Economic costs comprise the value of all outlays for vaccine introduction or screening and treatment, regardless of the source of funding, and take into account donated goods. For economic costs, the value of resources purchased by agencies other than the National Tanzanian Reproductive Health Programme, such as health personnel time costs and donated vaccines, are included. This analysis does not include the value of existing capital goods used to deliver services such as equipment when these are assumed to have excess capacity.

The current HPV vaccination module of the C4P tool has been improved compared to the version used in the previous exercise [4]. Depending on the objectives of the costing exercise, it can be used to either project costs in a 5-year period or retrospectively estimate cost expenditure in a 5-year period. Also, the tool can not only cost single-age cohort vaccination, but also multiple-age cohort vaccination, which is applicable to countries that newly introduce HPV vaccine for the recommended age group and for catch-up in an older age group.

The C4P tool was used to compare the alternative strategies and ultimately to focus on the detailed costing of the strategic plan [3]. The costs of cervical cancer prevention and control interventions were projected for a 5-year period, 2020–2024. The HPV vaccination module (version 4.1) is used to project costs of vaccinating 14-year-old girls while the screening and treatment module (version 47.6.5) project the cost of providing cervical cancer services for two target populations: (1) women living with HIV that start screening at 15 years and (2) women from the general population that start screening at 30 years. Table 3 shows the activities that are costed in the two modules.

**Table 3:** Inputs used in cervical cancer prevention and control services

Variable name	Assumption	Source	Variable name	Assumption	Source
Vaccination			Screening and treatment		
Target population	14-year-old girls; 670,905	National Bureau of Statistics	Target population	Women living with HIV aged 15–49 years: 7,852,280 Women in the general population aged 30–49 years: 5,802,963	World Population Prospects 2019
Vaccination site	Health facility: 40% At school: 55% Mobile outreach: 5%	Programme managers	% women assumed to be living with HIV	15–29 years: 3.1% 30–49 years: 10.5%	Tanzania HIV Impact Survey 2016–17 [9]
Coverage (second dose of 2-dose vaccine)	2020: 70% 2021: 72% 2022: 75% 2023: 80% 2024: 85%	Workshop expert opinion	Screening coverage	2020: 15% 2021: 20% 2022: 25% 2023: 30% 2024: 35%	Workshop expert opinion
Training	Council cascade and annual refreshers	Expert opinion	Epidemiology evaluation/quality assurance of the programme	HPV−: 90.2% HPV+/none: 1.6% Small lesions: 4.8% Large lesions: 0.4% Invasive cancer: 3.0%	Programme managers
Cold chain	No additional cold chain required	Programme managers	Rescreening # of years	HIV+: 3.0 years HIV−: 5.0 years	Programme managers
Health staff time	5 min nurse or medical attendant per vaccination	Workshop expert opinion	Health staff time		Workshop expert opinion
			HPV-DNA test	15 min (RN)	
			VIA	20 min each (RN, EN)	
			Cryotherapy	20 min each (RN, EN)	
			LEEP	35 min (specialist, MO, AMO)	
			CSDR	40 min each (oncologist, EN)	
			Chemotherapy	720 min oncologist, 2520 min (RN, EN)	
			Radiotherapy	135 min MO, 180 min (RN, PH), 280 min RO, 500 min RT	
			Palliative out/in	15 min AMO	
Local school visit transport	Per diem allowance of US$13 (30,000 TZA) and transport allowance	Programme managers	Existing equipment with excess capacity (not costed)	Waiting area, examination area, gynaecological examination couch	Workshop interviews
Vaccine price per dose	US$4.50 per dose	Gavi	Laboratory test prices	Not applicable	NHIF price schedule
Donations	80% of vaccine, injection supplies and shipping donated by Gavi	Gavi	Equipment prices	Not applicable	MDA, UNFPA, UNICEF, WHO
Assumptions relevant to both prevention and control services					
Exchange rate	2304 TZA to US$1	Oanda	Discount rate	7.0% (Bank of Tanzania)	Central bank rate

*AMO* assistant medical officer, *CSDR* comprehensive screening, diagnostic and referral, *DNA* deoxyribonucleic acid, *EN* enrolled nurse, *HIV* human immunodeficiency virus, *HPV* human papillomavirus, *LEEP* loop electrosurgical excision procedure, *MDA* Medical Device Authority, *MO* medical officer, *NHIF* National Health Insurance Fund, *PH* physicist, *RN* registered nurse, *RT* radiotherapist technician, *UNFPA* United Nations Population Fund, *VIA* visual inspection with acetic acid, *WHO* World Health Organization

Costing screening and treatment services is more complicated than vaccination since it comprises seven services rather than one: two for screening, two for treating pre-cancer and three for diagnosis and treatment of invasive cancer. The cost per cervical cancer service includes health personnel time, supply and materials, capital goods and other costs. The assumptions and data sources made in the estimation of vaccination, screening and treatment costs for each service are shown in Table 3.

All costs are reported in 2019 US dollars (US$).

## Data collection

The study team, comprising health economists, reproductive health specialists and epidemiologists, collected the data during two workshops in February and April 2019 in Dar Es Salaam with immunization and cervical cancer programme managers; the Ministry of Health, Community Development, Gender, Elderly and Children (MOHCDEC); and external partners (Joint United Nations Programme on HIV/AIDS, United States Center for Disease Control, Clinton Health Access Initiative, Johns Hopkins Program for International Education in Gynecology and Obstetrics, and London School of Hygiene and Tropical Medicine and WHO consultants). All participants attended the meetings in person. They collected information on time spent on service delivery using expert opinion, numbers of facilities/sites providing services, information on pricing and salaries and financial records on expenditures on planning, training, sensitization and monitoring as well as plans for scaling up service delivery.

## Results

### HPV vaccination costs

**Fig. 1:**
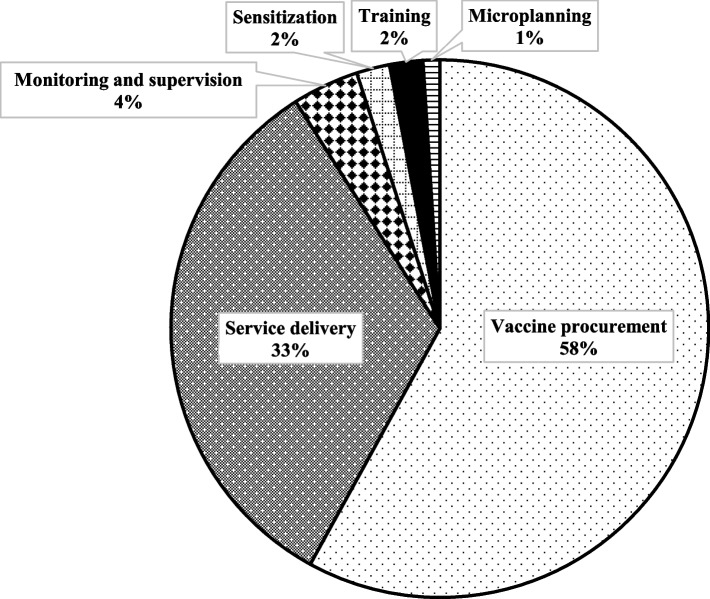
Composition of financial costs of HPV vaccine delivery in 2020–2024

Table 4 shows the projected costs of the HPV vaccination during 2020–2024. The total annual financial costs, i.e. the monetary outlays by the government, range from US$3.2 million in 2020 to US$4.1 million in 2024, while the annual economic costs range from US$8.3 million to US$10.8 million. Economic costs are higher than financial ones since these include the value of donated vaccines and existing health personnel salaries. The financial and economic costs per fully immunized girl (FIGs) are projected to be US$6.68 and US$17.31, respectively. The cost drivers for both financial and economic costs are vaccine and injection supply procurement (58.1% and 71.4%, respectively) and service delivery (33.4% and 24.0%, respectively) comprising personnel, per diem and transport (see Fig. 1 for a pie chart on financial cost distribution). Programmatic costs (including microplanning, training, sensitization, monitoring and supervision) account for about 8.4% of total financial costs and 4.5% of economic costs.

**Table 4:** Costing summary of HPV vaccination in 2020–2024 (2019 US dollars)

	2020	2021	2022	2023	2024	Total	Percentage
Coverage							
First dose	80%	82%	85%	87%	90%	85%	NA
Second dose	70%	72%	75%	80%	85%	77%	
Drop-out	12.5%	12.2%	11.8%	8%	5.6%	12.5%	
Financial							
Startup (US$000s) (annualized)							
Microplanning	25	25	25	25	25	125	0.7
Training	78	78	78	78	78	386	2.1
Sensitization	78	78	78	78	78	390	2.1
Recurrent (US$000s)							
Vaccine procurement^a^	1853	1962	2101	2261	2443	10,621	58.1
Service delivery	1066	1128	1208	1302	1405	6108	33.4
Monitoring and supervision	129	129	129	129	129	644	3.5
**Total financial costs (US$000s)**	**3229**	**3400**	**3619**	**3874**	**4158**	**18275**	**100%**
Economic							
Startup (US$000s) (annualized)							
Microplanning	30	30	30	30	30	138	0.3
Training	105	105	105	105	105	481	1.0
Sensitization	94	94	94	94	94	429	0.9
Recurrent (US$000s)							
Vaccine procurement	5899	6244	6688	7200	7775	33,806	71.4
Service delivery	1984	2100	2250	2422	2615	11,370	24.0
Monitoring and supervision	220	220	220	220	220	1102	2.3
**Total economic costs (US$000s)**	**8332**	**8793**	**9387**	**10,071**	**10,839**	**47,327**	**100%**
Number of FIGs	**469,634**	**497,998**	**534,646**	**588,497**	**644,036**	**2,734,810**	NA
Financial cost per FIG with vaccine costs	NA^b^	NA^b^	NA^b^	NA^b^	NA^b^	**$6.68**	NA
Economic cost per FIG with vaccine costs	NA^b^	NA^b^	NA^b^	NA^b^	NA^b^	**$17.31**	NA

*FIG* fully immunized girls; costs reported in 2019 US dollars (US$)

^a^Refers to 20% vaccine purchase and freight, handling and insurance charges

^b^Due to substantial one-time costs necessarily being spread over the 5 years and personnel costs being calculated per vaccination, the per FIG annual costs would not adequately reflect service volume. See instead the aggregate costs divided by the total numbers of fully immunized girls for the 5 years in the “Total” column

### Cervical cancer secondary and tertiary prevention programme costs

Table 5 shows the estimated unit cost per screening and treatment service based on the assumptions of the prices and quantities of resources needed. The service with the lowest cost is VIA screening, and the costliest one is radiotherapy cancer therapy.

**Table 5:** Cervical cancer services’ projected numbers provided and costs in 2020–2024 (2019 US dollars)

Screening and treatment service	Projected number of services provided in 5 years	Financial cost per service	Economic cost per service
Screening			
VIA	4,750,216	$3.66	$5.54
HPV-DNA testing	327,364	$9.20	$10.03
Pre-cancer			
Cryotherapy	117,549	$5.14	$7.21
LEEP	9796	$56.79	$70.03
Cancer			
Diagnosis	73,615	$100.65	$106.60
Chemotherapy	18,395	$292.56	$574.52
Radiotherapy	7361	$800.21	$893.80
Palliative care (outpatient)	9325	$7.05	$7.83
Palliative care (inpatient)	491	$33.57	$34.36

*DNA* deoxyribonucleic acid, *HPV* human papillomavirus, *LEEP* loop electrosurgical excision procedure, *VIA* visual inspection with acetic acid

Table 6 presents the projected costs of cervical cancer secondary and tertiary prevention programme costs during 2020–2024. The total cost of the programme over 5 years is projected to be US$49.9 million and US$78.20 million for financial and economic costs, respectively. Most of the projected costs are for service delivery (82% for financial costs and 72% for economic costs). The remaining projected financial and economic costs (18% and 28%, respectively) are for programmatic costs, i.e. monitoring and supervision, programme planning/microplanning, training and sensitization/social mobilization.

**Table 6:** Costing summary of cervical cancer secondary and tertiary prevention in 2020–2024 (2019 US dollars)

	2020	2021	2022	2023	2024	Total	Percentage
Coverage assumptions							
Screening	15%	20%	25%	30%	35%	N/A	N/A
^a^Drop-out for treatment if HPV+	50%	50%	50%	50%	50%	N/A	N/A
**Financial costs (000s)**							
**Service delivery**							
Screening	2712	3467	4119	4783	5333	20,413	40.9%
Pre-cancer	208	230	243	233	246	1161	2.3%
Diagnosis and cancer	3320	3816	4026	3865	4077	19,105	38.3%
**Programmatic support**							
Microplanning	62	62	62	62	62	310	0.6%
Training	64	64	64	64	64	320	0.6%
Sensitization/social mobilization	1505	1505	1505	1505	1505	7526	15.1%
Monitoring and supervision	204	204	204	204	204	1020	2.0%
**Total financial costs**	**8075**	**9348**	**10,223**	**10,717**	**11,491**	**49,853**	**100%**
**Economic costs (000s)**							
**Service delivery**							
Screening	4130	5120	6041	6779	7550	29,620	37.9%
Pre-cancer	276	305	321	308	324	1534	2.0%
Diagnosis and cancer	4458	5102	5328	5155	5394	25,427	32.5%
**Programmatic support**							
Microplanning	151	151	151	151	151	755	1.0%
Training	92	92	92	92	92	460	0.6%
Sensitization/social mobilization	3748	3748	3748	3748	3748	18,741	24.0%
Monitoring and supervision	330	330	330	330	330	1651	2.1%
**Total economic costs**	**13,185**	**14,848**	**16,011**	**16,562**	**17,579**	**78,423**	**100%**

^a^Drop-out rate assumes high loss to follow-up for the treatment of pre-cancer due partly to logistical difficulties for cryotherapy and LEEP and for invasive cancer due to the limited availability of tertiary health centres

Among all services, the largest share of projected costs is for screening, followed by diagnosis and cervical cancer treatment. The largest share of programmatic costs is for sensitization/social mobilization.

### Total costs

The total financial cost of comprehensive prevention and control over 5 years are projected to be US$67.6 million (annually US$11.2 to US$15.6 million), while total economic costs are projected to be US$123.6 million (annually US$21.1 to US$28.0 million) when all costs regardless of their source are included. The projected financial costs of screening and treatment are more than twice as costly as vaccination and 1.6 times as great as economic costs. The costliest service for financial costs is screening and pre-cancer treatment and vaccination for economic costs (due to the inclusion of donated vaccines) (Table 7).

**Table 7:** Total projected costs of cervical cancer prevention and control in 2020–2024 (2019 million US dollars)

Programme areas	2020	2021	2022	2023	2024	Total	Percentage
**Financial**							
Costs of HPV vaccination (including programmatic costs)	3.2	3.4	3.6	3.9	4.2	**18.3**	**27.1%**
Service delivery costs of screening and pre-cancer treatment	2.9	3.6	4.3	4.9	5.5	**21.2**	**31.4%**
Service delivery costs of cancer diagnosis, treatment and palliative care	3.3	3.8	4.0	3.9	4.1	**19.1**	**28.3%**
Programmatic costs of secondary and tertiary prevention	1.8	1.8	1.8	1.8	1.8	**9.2**	**13.3%**
**Total**	**11.2**	**12.6**	**13.7**	**14.5**	**15.6**	**67.6**	**100.0%**
**Economic**							
Costs of HPV vaccination (including programmatic costs)	8.3	8.8	9.4	10.	10.8	**47.3**	**38.3%**
Service delivery costs of screening and pre-cancer treatment	4.3	5.4	6.3	7.0	7.8	**30.8**	**24.9%**
Service delivery costs of cancer diagnosis, treatment and palliative care	4.5	5.1	5.3	5.2	5.4	**25.5**	**20.6%**
Programmatic costs of secondary and tertiary prevention	4.0	4.0	4.0	4.0	4.0	**20.0**	**16.2%**
**Total**	**21.1**	**23.3**	**25.0**	**26.6**	**28.0**	**123.6**	**100%**

## Discussion

The C4P tool allows programme managers working in low- and middle-income countries such as Tanzania to work with a study team and stakeholders to co-create a projection of the incremental costs of scaling up their programmes, for primary, secondary and tertiary prevention. The tool is a central part of a process that allows programme managers to consider the financial implications of alternative approaches to scaling up a cervical cancer programme. Furthermore, it acts as a catalyst for key stakeholders to engage in substantive discussions about how they can coordinate their efforts to avoid bottlenecks and gaps in the service delivery workflow. As the process of completing the tool progresses, programme managers, policymakers, donors and funders ideally will reach a convergence in their understanding of what needs to be done and how much it will cost to do it. This, in turn, aids champions of a cervical cancer programme to plan and advocate for resources for their programme so that they can reach their goals of reductions in mortality and morbidity and eventually elimination.

The process to estimate the costs of scaling up a comprehensive cervical cancer programme involves setting up a team of health economists, epidemiologists, clinicians and programme managers from both the immunization and the cancer units or programmes. Using the C4P tool effectively requires a trained health economist to be part of the study team and therefore involves training local health economists to use the tool. An additional benefit of the process is that the health system is strengthened since the capacity of local health economists in costing health programmes is improved.

The results of the pilot in Tanzania indicate that the cost driver for vaccination is vaccine procurement (58% and 71% of financial and economic costs, respectively). For non-vaccine costs, the cost driver is service delivery (33% and 24%, respectively), i.e. personnel salaries (for economic costs), transport and per diem. Programmatic activities (i.e. microplanning, training, sensitization and monitoring and evaluation) comprise a smaller proportion (8% of financial costs and 5% of economic costs) since these improve service demand and quality. The costliest programmatic activity is monitoring and supervision.

When the projected costs of vaccination are compared to the 2012 study by Hutubessy et al. in Tanzania [4], these decreased by 37% due to a change in the number of doses of HPV vaccines and a lower percentage of vaccinations taking place at schools. That is, the WHO recommended number of vaccine doses per girl has declined from three to two [8], leading to lower projected costs for procurement. In addition, in the 2012 study, it was assumed that all vaccines would be given at schools while this study assumes that approximately half of the target population would be vaccinated in health facilities and the other half in schools, leading to lower service delivery costs. Other projected costs have increased in this study, e.g. the cost per service delivery visit has increased due to higher per diems and travel allowances.

The projected financial annual costs of cervical cancer screening and treatment range from $8.0 million in 2020 to $11.4 million in 2024 while projected economic annual costs range from $13.1 million in 2020 to $17.5 million in 2024. The service with the highest share of costs is screening (combination of VIA and HPV-DNA testing) since the projected service volume is significantly higher than that for pre-cancer and cancer services even though the latter costs per service are much higher. Programmatic costs also comprise a large share, i.e. 16.3% and 25.6% for financial and economic costs, respectively.

To understand the context of implementing cervical cancer prevention and control in Tanzania, the proportion of the annual health budget that would be required to finance the programme was calculated. The estimated proportion of the annual 2020/2021 health budget of Tanzania [10] that would be required is 1.4%, and this suggests that a comprehensive prevention and control programme is likely to be affordable.

The costs of screening and treatment are similar to those found in other studies in low- and lower-middle-income countries. The cost of a woman screened with VIA in other studies US$3.33 [11] to US$3.67 [12] compares well with US$3.66 to US$5.44 in this study. The cost of a woman screened with HPV-DNA testing in other studies [US$6.27 to US$15.92] [13] is in the range of this study’s estimates [US$9.20 to US$10.03]. The estimate of a cryotherapy service in this study, US$5.14 to US$7.21, is lower, however, than in other studies, e.g. US$38 [11] or US$28.97 [12], possibly because the costs of equipment were calculated differently, i.e. these were divided by total national expected cases rather than by the number of cases referred to tertiary or referral hospitals. The costs of cancer services in this study, though, are similar to those found in Nelson et al. [14].

This study has some limitations. The first limitation of our study is that we illustrated the application of the C4P tool based only on the Tanzania Strategic Plan Cervical Cancer Prevention and Control [7], which had been developed before new WHO guidelines for cervical cancer prevention and control had been published. The C4P tool has the capability of comparing scenarios and could potentially have been used to calculate the costs of relevant alternative strategies such as 1-dose HPV vaccine regimes, self-collected samples for HPV testing and thermal ablation. The second limitation relates to the short 5-year study period of the C4P tool, which reflects its emphasis on programme management rather than policymaking. The impact of vaccination is realized only in the longer term, and so the significant cost savings of reductions in treatment for pre-cancer and for down-staged invasive cancer are not captured by the tool. The third limitation is that data collection did not include the sampling of health facilities due to the limited time frame and budget for the study. A final limitation of the C4P tool is that a formal uncertainty or sensitivity analysis is not included in the tool itself.

If Tanzania can scale up these services for the prevention and control of cervical cancer, there will be an important impact on cervical cancer morbidity and mortality over the medium and long term. The impact, though, will be affected by whether there is compliance with referrals and accurate diagnoses and effective treatment. So, it will be important during the scale-up not only to focus just on increasing the number of sites that provide services but also to ensure the quality of services. It will also be important to ensure the equity of service provision and access to screening and pre-cancer services at lower levels of the health system.

## Conclusions

The C4P tool modules for vaccination and screening and treatment can assist national cervical cancer programmes to estimate monetary resources needed as well as opportunity costs of reducing national cervical cancer incidence through primary, secondary and tertiary prevention.

## Data Availability

The C4P tool is publicly available at https://www.who.int/tools/who-cervical-cancer-prevention-and-control-costing-(c4p)-tool. The Tanzanian costing data is available upon request.

## Abbreviations

AIDS: Acquired immunodeficiency syndrome
AMO: Assistant medical officer
C4P: Cervical Cancer Prevention and Control Costing
CSDR: Comprehensive screening, diagnostic and referral
DNA: Deoxyribonucleic acid
EN: Enrolled nurse
FIG: Fully immunized girl
HIV: Human immunodeficiency virus
HPV: Human papillomavirus
IVD: Immunization and Vaccine Development Programme
LEEP: Loop electrosurgical excision procedure
MDA: Medical Device Authority
MO: Medical officer
MOHCDEC: Ministry of Health, Community Development, Gender, Elderly and Children
NHIF: National Health Insurance Fund
PH: Physicist
RN: Registered nurse
RT: Radiotherapist technician
SVA: Single visit approach strategy
UNFPA: United Nations Population Fund
UNJGP: United Nations Joint Global Programme
VIA: Visual inspection with acetic acid
WHO: World Health Organization
